# Safety criteria to start early mobilization in intensive care units. Systematic review

**DOI:** 10.5935/0103-507X.20170076

**Published:** 2017

**Authors:** Thais Martins Albanaz da Conceição, Ana Inês Gonzáles, Fernanda Cabral Xavier Sarmento de Figueiredo, Danielle Soares Rocha Vieira, Daiana Cristine Bündchen

**Affiliations:** 1 Hospital Universitário Polydoro Ernani de São Thiago), Universidade Federal de Santa Catarina - Florianópolis (SC), Brazil.; 2 Department of Physical Therapy, Universidade Federal de Santa Catarina - Araranguá, (SC), Brazil.

**Keywords:** Hospitalization, Rehabilitation, Respiration, artificial, Early ambulation, Critical care, Patient safety, Hospitalização, Reabilitação, Respiração artificial, Deambulação precoce, Cuidados críticos, Segurança do paciente

## Abstract

Mobilization of critically ill patients admitted to intensive care units should be performed based on safety criteria. The aim of the present review was to establish which safety criteria are most often used to start early mobilization for patients under mechanical ventilation admitted to intensive care units. Articles were searched in the PubMed, PEDro, LILACS, Cochrane and CINAHL databases; randomized and quasi-randomized clinical trials, cohort studies, comparative studies with or without simultaneous controls, case series with 10 or more consecutive cases and descriptive studies were included. The same was performed regarding prospective, retrospective or cross-sectional studies where safety criteria to start early mobilization should be described in the Methods section. Two reviewers independently selected potentially eligible studies according to the established inclusion criteria, extracted data and assessed the studies' methodological quality. Narrative description was employed in data analysis to summarize the characteristics and results of the included studies; safety criteria were categorized as follows: cardiovascular, respiratory, neurological, orthopedic and other. A total of 37 articles were considered eligible. Cardiovascular safety criteria exhibited the largest number of variables. However, respiratory safety criteria exhibited higher concordance among studies. There was greater divergence among the authors regarding neurological criteria. There is a need to reinforce the recognition of the safety criteria used to start early mobilization for critically ill patients; the parameters and variables found might contribute to inclusion into service routines so as to start, make progress and guide clinical practice.

## Abstract (pt)

Pacientes críticos internados em unidade de terapia intensiva devem ser mobilizados com base em critérios de segurança. O objetivo desta revisão foi verificar os critérios de segurança mais utilizados para iniciar a mobilização precoce em pacientes sob ventilação mecânica internados em unidade de terapia intensiva. Os artigos foram pesquisados nas bases de dados PubMed, PEDro, LILACS, Cochrane e CINAHL, tendo sido incluídos ensaios clínicos randomizados e controlados, ensaios clínicos quase randomizados, coortes, estudos comparativos com ou sem controles simultâneos, séries de casos com dez ou mais casos consecutivos, e estudos descritivos. O mesmo foi feito para estudos prospectivos, retrospectivos e transversais, nos quais, em sua metodologia, deveria constar a descrição dos critérios de segurança utilizados para iniciar a mobilização precoce. Dois revisores selecionaram, independentemente, estudos em potencial, de acordo com os critérios de inclusão, extraíram os dados e avaliaram a qualidade metodológica. Na análise dos dados, foi utilizada descrição narrativa para resumir as características e os resultados dos estudos obtidos, sendo os critérios de segurança categorizados nos seguintes subgrupos: cardiovasculares, respiratórios, neurológicos, ortopédicos e outros. Obtivemos 37 estudos elegíveis. O critério de segurança cardiovascular apresentou o maior número de variáveis identificadas. No entanto, o critério de segurança respiratório apresentou maior concordância. Houve maior divergência entre os autores em relação aos critérios neurológicos. Faz-se necessário reforçar o reconhecimento dos critérios de segurança utilizados para segurança da mobilização precoce do paciente crítico, ao mesmo tempo em que os parâmetros e as variáveis encontradas poderão auxiliar na incorporação à rotina dos serviços, com a intenção de iniciar, progredir e guiar a prática clínica.

## INTRODUCTION

The survival rates of the critically ill have increased in the past years; consequently, the number of morbidities such patients develop arising from long stays at the intensive care unit (ICU) has also increased.^(1-3)^ Within this context, early mobilization (EM) performed in a safe manner might reduce such deleterious effects.

Information on safety criteria for EM in adult ICUs were initially published by Stiller and Philips,^(4)^ followed by Stiller.^(5)^ Both studies were based on physiological principles and the authors' clinical experience. Gosselink et al.,^(1)^ together with the European Respiratory Society & European Society of Intensive Care Medicine, recommend that patient mobilization ought to be performed under adequate monitoring and with due safety. In turn, Hodgson et al.^(6)^ cited evidence provided by clinical studies and participants' consensus. Finally, Sommers et al.^(7)^ formulated evidence-based recommendations for effective and safe EM in the ICU setting.

Rehabilitation of ICU patients depends on various factors, such as previous physical strength and functioning, level of cooperation, devices connected and the prevalent mobilization culture in each individual service.^(8-10)^ Some studies have shown that EM is safe and feasible;^(11-13)^ however, there is not yet a consensus on its outcomes. Some studies^(3,6,13-17)^ have described potential benefits, such as reduction of the duration of mechanical ventilation (MV), length of stay in the ICU and the hospital, sedation and duration of delirium and hospital costs, in addition to improvement of the clinical and functional outcomes at hospital discharge. However, these results disagree with those from randomized controlled studies^(18-20)^ showing that early and intensive mobilization does not change patient functioning and quality of life either at discharge or 6 months after hospital discharge.

For outcomes to be favorable, knowledge of the relationship among potential benefits, eligibility for EM and its related adverse events are relevant.^(6,21)^ Even though the rate of adverse events is equal to or lower than 4%,^(14,22-25)^ patients need to be thoroughly assessed based on safety criteria before starting EM.^(6)^ Yet, the safety criteria used vary among different types of ICUs. As a function of this lack of standardization of safety criteria, there is no consensus on which should be used to start EM so as to minimize risk. To provide increasingly more consistent grounds for clinical practice, the aim of the present study was to establish, by means of a systematic review, the most widely used safety criteria to start EM for patients under MV and admitted to the ICU.

## METHODS

The present systematic review followed the recommendations formulated in Preferred Reporting Items for Systematic Reviews and Meta-Analyses (PRISMA).^(26)^

### Inclusion criteria

The following types of studies were included: randomized clinical trials, prospective and retrospective studies, case series with at least 10 consecutive patients and studies with independent or parallel group design. Determination of design followed the classification formulated by the Cochrane Collaboration.^(27)^ Randomized clinical trial protocols and care delivery protocols were also included. Patients had to be over 18 years old, admitted to the ICU and under MV for more than 24 hours. Articles in Portuguese, English, Spanish and French were included. Articles had to contain, in the Methods section, a description of the safety criteria used to start EM.

### Exclusion criteria

Articles in which safety criteria to start EM in patients admitted to the ICU and under MV were not described were excluded. In addition, review studies, monographs/dissertations/theses, annals, chapters from books and experts' points of view or opinions were excluded.

### Search strategy

The search was independently performed by two investigators in the PubMed, Physiotherapy Evidence Database (PEDro), Literatura Latino-Americana e do Caribe em Ciências da Saúde (LILACS; in English: Latin American and Caribbean Health Sciences Literature), Cochrane and Cumulative Index to Nursing and Allied Health Literature (CINAHL) electronic databases from the time the databases were launched to May 2015. As per the review aims, the search followed PRISMA recommendations^(26)^ and considered the concepts of target patient and intervention of the PICO strategy, i.e., concepts control and outcome were not included in the search strategy. Outcomes were not defined as search criteria.

Based on Medical Subject Heading (MeSH) terms and adequate descriptors and Boolean operators, the initial search was performed in the PubMed database as follows: [(intensive care units/or intensive care.tw or critical illness/) and (early ambulation/or early mobilization.tw or passive mobilization or active mobilization)]. The search strategy for the other databases was modified as per individual specificities; these details can be requested from the authors. To complement the electronic search, a manual search was performed based on the references cited in the included articles.

### Study selection

Two investigators independently conducted a search for potentially eligible studies. Articles were first categorized according to title. Next, their abstracts were analyzed, and only potentially eligible articles were selected. Cases of disagreement were solved by a third examiner, who made the final decision on the eligibility of such articles.

### Methodological quality

Randomized clinical trials were assessed according to the PEDro scale,^(28)^ which consists of 11 items to evaluate a study's methodological quality (internal validity and statistical information). With the exception of the first, each item with an affirmative answer was attributed a score of 1 in the final overall classification (score: 0 to 10). Studies with scores of 7 to 10 were considered as high quality, 5 to 6 as intermediate quality and 0 to 4 as low quality.^(29)^ It should be noted that the PEDro score was not used as an inclusion or exclusion criterion but as an indicator of the quality of the scientific evidence provided in the included articles.

### Data extraction and variable selection

Data relative to safety criteria were independently extracted from each eligible study by two examiners and recorded on a standardized data extraction form. The safety criteria were categorized as cardiovascular, respiratory, neurological, orthopedic and other; the corresponding variables and parameters were entered in a specific form. Regarding the variables relative to each safety criteria, only the ones cited in at least three articles were considered.

## RESULTS

**Figure 1:**
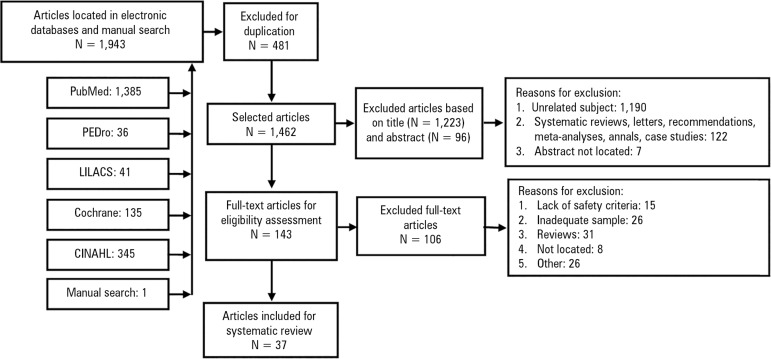
Flowchart of the search process

A total of 1,943 articles were located, and 1,462 were selected for triage. A total of 1,223 articles were excluded based on their titles and 96 additional studies based on their abstracts. A total of 143 articles were selected for full-text analysis. Finally, 37 studies were included for systematic review, as they met the inclusion and exclusion criteria (Figure 1).

The sample size varied from 11 to 2,176 participants, for a total of 6,641 patients from both genders, with an age range of 45.2 to 75.2 years old, and admitted to clinical, surgical or general ICU.

Table 1 describes the methodological quality of the randomized clinical trials.^(9,13,23,30-32)^ Three out of six studies were registered in PEDro,^(9,13,30)^ and the corresponding score was available in the database. The other three studies^(23,31,32)^ were scored based on full-text analysis and examiner consensus. Scores varied from 4 to 8. No study was scored on the items related to patient and therapist blinding; in one single study, assessors were blinded.^(9)^ Two studies exhibited the minimum score, 4,^(30,32)^ and only Schweickert et al.'s^(9)^ study had a score of 8.

**Table 1:** Methodological classification of articles according to the PEDro scale

Criteria	Schweickert et al.^(9)^	Collings et al.^(13)^	Médrinal et al.^(23)^	Nava^(30)^	Dantas et al.^(31)^	Dong et al.^(32)^
Eligibility criteria *	Yes	Yes	Yes	Yes	Yes	Yes
Random allocation	Yes	Yes	Yes	Yes	Yes	Yes
Concealed allocation	Yes	No	Yes	No	No	No
Homogeneity at baseline	Yes	Yes	No	Yes	Yes	Yes
Subject blinding	No	No	No	No	No	No
Therapist blinding	No	No	No	No	No	No
Assessor blinding	Yes	No	No	No	No	No
Adequate follow up	Yes	Yes	Yes	No	Yes	No
Intention to treat	Yes	Yes	Yes	No	Yes	Yes
Comparison between groups	Yes	Yes	Yes	Yes	Yes	Yes

* Not included in the final score.

The safety criteria to start EM are described in table 2. As is shown, the cardiovascular criteria exhibited the largest number of variables (9 total), among which absence of myocardial ischemia, absence of arrhythmia and hemodynamic stability stood out. None of the selected studies reported parameters for tolerated dose of vasoactive drugs or drug combination to attain hemodynamic stability; therefore, these variables could not be quantified.

**Table 2:** Located safety criteria with corresponding categories, variables, parameters and references

Criteria	Variables	Parameters	References
Cardiovascular	Heart rate	> 40bpm and < 130bpm	Pohlman et al.,^(2)^ Davis et al.,^(24)^ Schweickert et al.,^(9)^ Dong et al.,^(32)^ Brummel et al.^(33)^ and Harris et al.^(34)^
	Systolic arterial pressure	< 180mmHg	Brummel et al.,^(33)^ Harris et al.^(34)^ e Dammeyer et al.^(36)^
		> 90mmHg and < 200mmHg	Davis et al.,^(24)^ Schweickert et al.,^(9)^ Dantas et al.,^(31)^ Timmerman,^(37)^ Lee et al.^(25)^ and Bourdin et al.^(38)^
	Mean arterial pressure	> 60mmHg	Dammeyer et al.,^(36)^ Segers et al.^(39)^ and Engel et al.^(40)^
		> 60mmHg and < 110mmHg	Perme et al.,^(12)^ Perme et al.,^(35)^ and Mah et al.^(41)^
		> 65mmHg < 110mmHg	Davis et al.,^(24)^ Schweickert et al.,^(9)^ Dong et al.^(32)^ and Lee et al.^(25)^
	Hemodynamic stability	--	Clark et al.,^(11)^ Collings et al.,^(13)^ Perme et al.,^(35)^ Engel et al.,^(40)^ Mah et al.,^(41)^ Dickinson et al.^(42)^ and Titsworth et al.^(43)^
	No vasoactive drugs	--	Bourdin et al.,^(38)^ Ronnebaum et al.,^(44)^ Thomsen et al.^(45)^ and Bailey et al.^(10)^
	No increase of vasopressor dose in the past 2 hours	--	Davis et al.,^(24)^ Needham et al.,^(14)^ Brummel et al.,^(33)^ Needham et al.^(46)^ and Balas et al.^(47)^
	No myocardial ischemia	--	Pohlman et al.,^(2)^ Needham et al.,^(14)^ Schweickert et al.,^(9)^ Dammeyer et al.,^(36)^ Balas et al.,^(47)^ Wang et al.,^(48)^ Berney et al.^(49)^ and Drolet et al.^(50)^
	No arrhythmia	--	Abrams et al.,^(21)^ Nava,^(30)^ Dammeyer et al.,^(36)^ Timmerman,^(37)^ Lee et al.,^(25)^ Dickinson et al.,^(42)^ Wang et al.,^(48)^ Berney et al.^(49)^ and Drolet et al.^(50)^
	No femoral artery catheter	--	Clark et al.,^(11)^ Brummel et al.^(33)^ and Timmerman^(37)^
	No repetition of antiarrhythmic agent	--	Needham et al.,^(14)^ Balas et al.^(47)^ and Drolet et al.^(50)^
Respiratory	Respiratory rate	> 5bpm < 40bpm	Pohlman et al.,^(2)^ Davis et al.,^(24)^ Schweickert et al.,^(9)^ Médrinal et al.,^(23)^ Dong et al.,^(32)^ Brummel et al.,^(33)^ Harris et al.,^(34)^ Dammeyer et al.^(36)^ and Olkowski et al.^(51)^
		< 35bpm	Timmerman,^(37)^ Lee et al.,^(25)^ Bourdin et al.,^(38)^ Wang et al.,^(48)^ Berney et al.^(49)^ and Drolet et al.,^(50)^
	Peripheral oxygen saturation	> 88%	Pohlman et al.,^(2)^ Davis et al.,^(24)^ Perme et al.,^(12)^ Needham et al.,^(14)^ Schweickert et al.,^(9)^ Dong et al.,^(32)^ Brummel et al.,^(33)^ Harris et al.,^(34)^ Dammeyer et al.,^(36)^ Drolet et al.^(50)^ and Olkowski et al.^(51)^
		≥ 90%	Collings et al.,^(13)^ Dantas et al.^(31)^ e Médrinal et al.^(23)^
	Mechanical ventilation parameters	FiO2 < 0.6 and/or PEEP < 10cmH2O	Perme et al.,^(12)^ Collings et al.,^(13)^ Needham et al.,^(14)^ Dantas et al.,^(31)^ Médrinal et al.,^(23)^ Brummel et al.,^(33)^ Harris et al.,^(34)^ Perme et al.,^(35)^ Timmerman,^(37)^ Lee et al.,^(25)^ Segers et al.,^(39)^ Balas et al.,^(47)^ Wang et al.^(48)^ and Drolet et al.^(50)^
		FiO2 ≤ 0.6 and PEEP ≤ 10cmH2O	Davis et al.,^(24)^ Mah et al.,^(41)^ Dickinson et al.,^(42)^ Thomsen et al.,^(45)^ Bailey et al.^(10)^ and Needham et al.^(46)^
	Airway protection	-	Pohlman et al.,^(2)^ Schweickert et al.,^(9)^ Brummel et al.^(33)^ and Dammeyer et al.^(36)^
Neurological	Intracranial pressure	Not elevated	Pohlman et al.,^(2)^ Schweickert et al.,^(9)^ Dantas et al.,^(31)^ Brummel et al.,^(33)^ Dammeyer et al.,^(36)^ Titsworth et al.^(43)^ and Meyer et al.^(53)^
	Level of consciousness	Not in coma	Davis et al.,^(24)^ Thomsen et al.,^(45)^ Bailey et al.^(10)^ and Witcher et al.^(52)^
		No agitation	Médrinal et al.,^(23)^ Harris et al.,^(34)^ Bourdin et al.^(38)^ and Segers et al.^(39)^
		Understands and performs commands correctly	Nava,^(30)^ Perme et al.,^(35)^ Bourdin et al.,^(38)^ Thomsen et al.^(45)^ and Wang et al.^(48)^
Cardiovascular	Heart rate	> 40bpm and < 130bpm	Pohlman et al.,^(2)^ Davis et al.,^(24)^ Schweickert et al.,^(9)^ Dong et al.,^(32)^ Brummel et al.^(33)^ and Harris et al.^(34)^
	Systolic arterial pressure	< 180mmHg	Brummel et al.,^(33)^ Harris et al.^(34)^ e Dammeyer et al.^(36)^
		> 90mmHg and < 200mmHg	Davis et al.,^(24)^ Schweickert et al.,^(9)^ Dantas et al.,^(31)^ Timmerman,^(37)^ Lee et al.^(25)^ and Bourdin et al.^(38)^
	Mean arterial pressure	> 60mmHg	Dammeyer et al.,^(36)^ Segers et al.^(39)^ and Engel et al.^(40)^
		> 60mmHg and < 110mmHg	Perme et al.,^(12)^ Perme et al.,^(35)^ and Mah et al.^(41)^
		> 65mmHg < 110mmHg	Davis et al.,^(24)^ Schweickert et al.,^(9)^ Dong et al.^(32)^ and Lee et al.^(25)^
	Hemodynamic stability	--	Clark et al.,^(11)^ Collings et al.,^(13)^ Perme et al.,^(35)^ Engel et al.,^(40)^ Mah et al.,^(41)^ Dickinson et al.^(42)^ and Titsworth et al.^(43)^
	No vasoactive drugs	--	Bourdin et al.,^(38)^ Ronnebaum et al.,^(44)^ Thomsen et al.^(45)^ and Bailey et al.^(10)^
	No increase of vasopressor dose in the past 2 hours	--	Davis et al.,^(24)^ Needham et al.,^(14)^ Brummel et al.,^(33)^ Needham et al.^(46)^ and Balas et al.^(47)^
	No myocardial ischemia	--	Pohlman et al.,^(2)^ Needham et al.,^(14)^ Schweickert et al.,^(9)^ Dammeyer et al.,^(36)^ Balas et al.,^(47)^ Wang et al.,^(48)^ Berney et al.^(49)^ and Drolet et al.^(50)^
	No arrhythmia	--	Abrams et al.,^(21)^ Nava,^(30)^ Dammeyer et al.,^(36)^ Timmerman,^(37)^ Lee et al.,^(25)^ Dickinson et al.,^(42)^ Wang et al.,^(48)^ Berney et al.^(49)^ and Drolet et al.^(50)^
	No femoral artery catheter	--	Clark et al.,^(11)^ Brummel et al.^(33)^ and Timmerman^(37)^
	No repetition of antiarrhythmic agent	--	Needham et al.,^(14)^ Balas et al.^(47)^ and Drolet et al.^(50)^
Respiratory	Respiratory rate	> 5bpm < 40bpm	Pohlman et al.,^(2)^ Davis et al.,^(24)^ Schweickert et al.,^(9)^ Médrinal et al.,^(23)^ Dong et al.,^(32)^ Brummel et al.,^(33)^ Harris et al.,^(34)^ Dammeyer et al.^(36)^ and Olkowski et al.^(51)^
		< 35bpm	Timmerman,^(37)^ Lee et al.,^(25)^ Bourdin et al.,^(38)^ Wang et al.,^(48)^ Berney et al.^(49)^ and Drolet et al.,^(50)^
	Peripheral oxygen saturation	> 88%	Pohlman et al.,^(2)^ Davis et al.,^(24)^ Perme et al.,^(12)^ Needham et al.,^(14)^ Schweickert et al.,^(9)^ Dong et al.,^(32)^ Brummel et al.,^(33)^ Harris et al.,^(34)^ Dammeyer et al.,^(36)^ Drolet et al.^(50)^ and Olkowski et al.^(51)^
		≥ 90%	Collings et al.,^(13)^ Dantas et al.^(31)^ e Médrinal et al.^(23)^
	Mechanical ventilation parameters	FiO2 < 0.6 and/or PEEP < 10cmH2O	Perme et al.,^(12)^ Collings et al.,^(13)^ Needham et al.,^(14)^ Dantas et al.,^(31)^ Médrinal et al.,^(23)^ Brummel et al.,^(33)^ Harris et al.,^(34)^ Perme et al.,^(35)^ Timmerman,^(37)^ Lee et al.,^(25)^ Segers et al.,^(39)^ Balas et al.,^(47)^ Wang et al.^(48)^ and Drolet et al.^(50)^
		FiO2 ≤ 0.6 and PEEP ≤ 10cmH2O	Davis et al.,^(24)^ Mah et al.,^(41)^ Dickinson et al.,^(42)^ Thomsen et al.,^(45)^ Bailey et al.^(10)^ and Needham et al.^(46)^
	Airway protection	-	Pohlman et al.,^(2)^ Schweickert et al.,^(9)^ Brummel et al.^(33)^ and Dammeyer et al.^(36)^
Neurological	Intracranial pressure	Not elevated	Pohlman et al.,^(2)^ Schweickert et al.,^(9)^ Dantas et al.,^(31)^ Brummel et al.,^(33)^ Dammeyer et al.,^(36)^ Titsworth et al.^(43)^ and Meyer et al.^(53)^
	Level of consciousness	Not in coma	Davis et al.,^(24)^ Thomsen et al.,^(45)^ Bailey et al.^(10)^ and Witcher et al.^(52)^
		No agitation	Médrinal et al.,^(23)^ Harris et al.,^(34)^ Bourdin et al.^(38)^ and Segers et al.^(39)^
		Understands and performs commands correctly	Nava,^(30)^ Perme et al.,^(35)^ Bourdin et al.,^(38)^ Thomsen et al.^(45)^ and Wang et al.^(48)^
		Opens eyes in response to verbal stimulus	Davis et al.,^(24)^ Needham et al.,^(14)^ Olkowski et al.^(51)^ and Engel et al.^(40)^
		Responds to verbal stimulus	Collings et al.,^(13)^ Mah et al.^(41)^ and Bailey et al.^(10)^
	No neurological and/or neuromuscular diseases hindering mobilization	--	Pohlman et al.,^(2)^ Dantas et al.,^(31)^ Segers et al.,^(39)^ Engel et al.,^(40)^ Ronnebaum et al.,^(44)^ Meyer et al.^(53)^ Winkelman et al.^(54)^ and Hopkins et al.^(55)^
Orthopedic	No unstable fracture	--	Clark et al.,^(11)^ Dantas et al.,^(31)^ Timmerman,^(37)^ Engel et al.^(40)^ and Meyer et al.^(53)^
	No bone instability	--	Clark et al.,^(11)^ Titsworth et al.^(43)^ and Witcher et al.^(52)^
	No orthopedic contraindications to mobilization	--	Collings et al.,^(13)^ Nava^(30)^ and Drolet et al.^(50)^
Other	No neuromuscular blocking agent	--	Abrams et al.,^(21)^ Timmerman,^(37)^ Segers et al.^(39)^ and Witcher et al.^(52)^
	No open abdomen	--	Clark et al.,^(11)^ Engel et al.,^(40)^ Balas et al.^(47)^ and Hopkins et al.^(55)^
	Not under palliative care	--	Pohlman et al.,^(2)^ Médrinal et al.,^(23)^ Segers et al.,^(39)^ Engel et al.,^(40)^ Titsworth et al.,^(43)^ Meyer et al.^(53)^ and Hopkins et al.^(55)^
	No deep vein thrombosis	--	Collings et al.,^(13)^ Needham et al.,^(14)^ Lee et al.^(25)^ and Drolet et al.^(50)^
	Not under continuous hemodialysis	--	Schweickert et al.,^(9)^ Dammeyer et al.,^(36)^ Bourdin et al.^(38)^ and Titsworth et al.^(43)^
	Body temperature	< 38.5°	Collings et al.,^(13)^ Segers et al.,^(39)^ Wang et al.^(48)^ and Berney et al.^(49)^
	No active gastrointestinal bleeding	--	Pohlman et al.,^(2)^ Schweickert et al.,^(9)^ Brummel et al.^(33)^ and Dammeyer et al.^(36)^
	No active bleeding	--	Abrams et al.,^(21)^ Timmerman,^(37)^ Lee et al.^(25)^ and Engel et al.^(40)^

FiO_2_ - fraction of inspired oxygen; PEEP - positive end-expiration pressure

Relative to the respiratory criteria, variables related with MV - fraction of inspired oxygen (FiO_2_) < 0.6 and/or positive end-expiratory pressure (PEEP) < 10cmH_2_O - were the ones with highest concordance, being cited by 14 authors.

As concerns the neurological criteria, the patients' level of consciousness was subjectively assessed. Therefore, this variable exhibited greater variation.

Table 3 describes information on study design, sample characteristics, ICU type, mobilization protocols and occurrence of adverse events. Most were general ICUs (14) followed by 8 clinical ICUs. The mobilization protocols were similar regarding the treatment offered; a large part of the studies followed a same order of progression: mobilization in bed, sitting on the edge of bed, standing and walking. The safety of these interventions was assessed based on the occurrence of adverse events. Although 15 studies did not report on this outcome, the rate of adverse events was low. When mentioned, the most frequent adverse events were desaturation, tachypnea, heart rate changes, loss of devices (such as tubes and catheters) and postural hypotension.

**Table 3:** Design of selected studies, intensive care unit type, mobilization protocol and description of adverse events

Study type	Reference	Country	N	ICU type	Mobilization protocol	Adverse events
Randomized clinical trial	Collings et al.^(13)^	United Kingdom	11	General	Sitting on the edge of the bed and passive chair transfer	Two AEs: desaturation due to ventilator circuit condensation ^(1)^ and HR elevation above 80% of the upper HR limit before mobilization ^(1)^
Randomized clinical trial	Schweickert et al.^(9)^	United States	104	Clinical	Passive, active-assisted and active mobilization, sitting on the edge of the bed, activities of daily living training, transfer, standing, walking	Two AEs: desaturation below 80% and loss of radial artery catheter
Randomized clinical trial	Dong et al.^(32)^	China	60	General	Active mobilization, sitting on the edge of the bed, transfer, standing and walking	One AE: postural hypotension
Randomized clinical trial	Médrinal et al.^(23)^	France	12	General	Passive mobilization and sitting on the edge of the bed	AEs in less than 3% of interventions
Randomized clinical trial	Dantas et al.^(31)^	Brazil	59	General	Positioning, stretching, passive mobilization, active-assisted exercise, sitting on the edge of the bed, resistance training, ergometric bicycle, transfer, balance training and walking	Not reported
Randomized clinical trial	Nava^(30)^	Italy	80	Respiratory	Passive and active mobilization, sitting on the edge of the bed, transfer, respiratory muscle training specific exercises, ergometric bicycle and walking	Not reported
Prospective study	Balas et al.^(47)^	United States	296	General	No protocol; authors recorded whether patients performed daily physical therapy and were mobilized out of bed	Seven cases of unplanned extubation (p = 0.98)
Partly prospective, partly retrospective study	Needham et al.^(14)^	United States	57	Clinical	Transfer, sitting on the edge of the bed, standing and walking	Four AEs, not characterized
Retrospective study	Dickinson et al.^(42)^	United States	1,112	Surgical	Passive and active mobilization, positioning, sitting on the edge of the bed, standing, chair transfer and walking with or without support	Not reported
Retrospective study	Ronnebaum et al.^(44)^	United States	28	General	Passive and active mobilization in bed, stretching, transfer, gait training	None
Retrospective study	Abrams et al.^(21)^	United States	35	Clinical	Passive and active-assisted mobilization in bed, positioning, sitting on the edge of the bed, transfer, standing, marching in place and ambulation	Not reported
Retrospective study	Witcher et al.^(52)^	United States	68	Neurological	Passive and active mobilization, sitting on the edge of the bed, standing and walking	Not reported
Retrospective study	Clark et al.^(11)^	United States	2,176	Trauma and burns	Passive mobilization, sitting on the edge of the bed, active exercise, transfer, walking	None
Retrospective study	Olkowski et al.^(51)^	United States	25	Neurosurgical	Positioning, education program, functional training and therapeutic exercise	AEs in 5.9% of sessions; MAP < 70 mmHg (9 patients), MAP > 120 mmHg (7 patients) and HR > 130 bpm (1 patient)
Retrospective study	Lee et al.^(25)^	Korea	99	Clinical	Neuromuscular electrical stimulation, passive and active mobilization, mobilization in bed, transfer, standing, therapeutic exercise and walking	26 potential AEs (5%; 95%CI 3.4-7.3%) in 17 patients (17.2%; 95%CI 10.6-26.4%). ECMO use was independently associated with AEs, OR 5.8 (95%CI 2.2-15.6, p < .001)
Retrospective study	Engel et al.^(40)^	United States	294	General	Mobilization, standing, chair transfer, gait training	Accidental device loss. Not quantified
Case series	Winkelman et al.^(54)^	United States	19	General	No specific protocol. Passive mobilization, sitting out of bed and walking were considered as therapeutic activity	Not reported
Case series	Segers et al.^(39)^	Belgium	50	General	Neuromuscular electrical stimulation	None
Case series	Pohlman et al.^(2)^	United States	49	General	Passive, active-assisted and active mobilization, sitting on the edge of the bed, balance training, standing, marching in place and ambulation	AEs in 16% of sessions (80/498). Desaturation (6%), HR elevation over 20% (4.2%), asynchrony/tachypnea (4%), agitation/discomfort (2%) and device loss (0.8%)
Case series	Drolet et al.^(50)^	United States	426	General	Education program, walking with or without aids	Not reported
Case series	Davis et al.^(24)^	United States	230	General	Education program, positioning in bed, mobilization in bed training, transfer and therapeutic exercise	1 AE/171 sessions: postural hypotension
Case series	Thomsen et al.^(45)^	United States	104	Respiratory	Sitting on the edge of the bed, chair transfer, functional activities, walking with walker and/or with or without additional aids	Not reported
Case series	Hopkins et al.^(55)^	United States	72	Respiratory	Passive and active mobilization, sitting on the edge of the bed, transfer and walking	Not reported
Case series	Harris et al.^(34)^	United States	21	Cardiological	Passive and active mobilization, sitting on the edge of the bed, transfer and walking	Not reported
Case series	Perme et al.^(35)^	United States	77	Cardiovascular	Sitting on the edge of the bed, chair transfer and walking	None
Case series	Titsworth et al.^(43)^	United States	170	Clinical	Positioning, passive and active mobilization, sitting on the edge of the bed, transfer, standing and walking	None
Case series	Bourdin et al.^(38)^	France	20	Clinical	Mobilization in and out of bed, transfer with and without support, walking	AEs in 3% of sessions (13/424): desaturation (< 88%) for more than 1 minute (4 patients), unplanned extubation (1 patient), postural hypotension (1 patient ) and muscle tone drop (7 patients)
Case series	Bailey et al.^(10)^	United States	103	Respiratory	Sitting on the edge of the bed and out of bed, walking	AEs in less than 1% of activities (14/1,449); most frequent: falls without injury, hypotension, desaturation, displacement of gastric feeding tube and one episode of hypertension
Case series	Berney et al.^(49)^	Australia	74	General	Mobilization in bed, marching in place, sitting-rising up training and walking	None
Independent group design	Wang et al.^(48)^	Australia	33	General	Passive mobilization, mobilization in bed, standing (with and without support) and marching in place	None
Independent group design	Mah et al.^(41)^	United States	59	Surgical	Passive and active mobilization, sitting on the edge of the bed, standing, chair transfer and walking	None
Randomized clinical trial protocol	Brummel et al.^(33)^	United States	-	-	Passive mobilization, sitting on the edge of the bed, standing, walking and activities of daily living training	Not reported
Randomized clinical trial protocol	Meyer et al.^(53)^	United States	200	Surgical	Positioning, passive and active mobilization, sitting on the edge of the bed, transfer, standing and walking	Not reported
Care delivery protocol	Timmerman^(37)^	United States	-	-	Passive mobilization, sitting on the edge of the bed, standing, chair transfer and walking	Not reported
Care delivery protocol	Perme et al.^(12)^	United States	-	-	Education, positioning, mobilization in the bed, transfer, walking and therapeutic exercise	Not reported
Care delivery protocol	Dammeyer et al.^(36)^	United States	388	Clinical	Activities in bed, sitting on the edge of the bed, marching in place and ambulation	Not reported
Care delivery protocol	Needham et al.^(46)^	United States	30	Clinical	Passive and active mobilization, sitting on the edge of the bed, transfer and walking	AEs in 1% of sessions, not specified

ICU - intensive care unit; AE - adverse event; HR - heart rate; PAM - mean arterial pressure; CI - confidence interval; ECMO - extracorporeal membrane oxygenation; OR - odds ratio.

## DISCUSSION

The present study stands out for having systematically assessed the safety criteria most widely employed to start EM for critically ill patients under MV and admitted to the ICU according to their individual clinical condition and the invasive devices connected to them.

According to the literature, prolonged immobilization of critically ill patients has negative repercussions on the musculoskeletal, cardiovascular and respiratory systems, the skin and cognition.^(41,56)^ To prevent and minimize such effects, immediate physical therapy intervention is necessary, provided the patient exhibits the clinical stability needed to meet the vascular and oxygen demands posed by this type of intervention.^(7,57)^

Cardiovascular criteria were the most often cited; this finding might be accounted for by the fact that upon being stimulated, bedridden patients with a long stay at the hospital require additional cardiovascular work to maintain their blood pressure, cardiac output and adequate and constant cerebral blood flow.^(58)^ On these grounds, hemodynamically unstable patients who require high doses of vasopressors are not fit to start or advance in the therapy.^(21)^ The same was the case for the results corresponding to hemodynamic stability, mentioned in seven studies, and the lack of use of vasoactive drugs, cited by four authors.

Specifically concerning devices inserted on the femoral region, the observational study by Perme et al.^(35)^ demonstrated the safety of mobilization based on a large number of sessions (210) and performed activities (630). Presence of a femoral catheter is no reason to restrict this practice, as it is no longer a contraindication for mobilization of the critically ill.^(35)^ Stiller^(5)^ observed that mobilization might be limited by the devices connected to patients. However, there is disagreement regarding patients subjected to hemodialysis; five among the studies included in the present review contraindicate mobilization in such cases. In contrast, Hodgson et al.^(6)^ and Wang et al.^(48)^ assert that mobilization of patients in the ICU setting is safe and feasible. Finally, Wang et al.^(48)^ conclude that intervention does not cause displacement, hematoma or bleeding, while successive interruptions might interfere with the outcomes of therapy.

The respiratory criteria exhibited higher concordance among the included studies. In this regard, we emphasize peripheral oxygen saturation (SpO_2_), mentioned in 14 studies, 11 of which consider SpO_2_ > 88% safe to start mobilization. According to Stiller and Philips^(4)^ and Amidei et al.,^(59)^ SpO_2_ is a safe and individualized monitoring parameter to incorporate into clinical practice. This finding is similar to the ones reported by Stiller et al.^(5)^ and Gosselink et al.,^(1)^ according to whom SpO_2_ > 90% with 4% oscillation is indicative of satisfactory respiratory reserve to tolerate mobilization.

As a function of the need for MV in critically ill patients, they are benefited by advances in intensive care and new approaches to MV.^(39)^ The feasibility and safety of mobilization of patients with artificial airways have already been demonstrated, provided the latter are secured and in their proper place.^(12)^ Twenty studies mentioned ventilation parameters; 14 of them cited FiO_2_ < 0.6 and/or PEEP < 10 cmH_2_O. FiO_2_ < 0.6 was also selected by Gosselink et al.^(60)^ as a criterion to start their mobilization protocol. Similar parameters are recommended by Hodgdon et al.^(6)^ and Sommers et al.,^(7)^ who consider FiO_2_ ≤ 0.6 and PEEP ≤ 10 cmH_2_O to be safe for mobilization of the critically ill.

Among the neurological criteria, assessments of intracranial pressure (ICP) and level of consciousness stood out. Witcher et al.^(52)^ considered that patients with elevated IPC and in whom deep sedation is combined with neuromuscular blockers are not candidates for participation in EM protocols and daily sedation interruption. Other reasons hindering EM are paralysis or paresis, cognitive dysfunction and abnormal brain perfusion, in addition to the use of devices for continuous brain monitoring.^(17,52)^

Regarding continuous monitoring of the patients' level of consciousness, daily interruption of sedation or maintenance of the minimally required levels are recommended to enable a more trustworthy assessment, in addition to reducing the severity of complications associated with stays in the ICU.^(9,46)^ The present systematic review found that the patients' level of consciousness was not assessed in an objective manner, with the help of scales, but subjectively, resulting in a wide variation of parameters. This finding might be explained by the various aims and methods of the studies; some of them required patients to be awake and cooperate with the treatment suggested, while in others, patients were under deep sedation.

Adverse events are usually associated with respiratory or cardiovascular complications and with the devices connected to patients.^(25)^ Collings et al.^(13)^ asserted that such events are a reflection of the limited individual reserve of patients and might manifest the physiological changes expectably induced by exercise.^(2)^ Adverse events do not increase hospital costs or length of stay at the hospital.^(13)^

Some findings do not reflect the situation in clinical practice. Patients under palliative care are often not included in study populations due to their extreme frailty and lack of chances for a cure with treatment and their consequent higher odds for treatment not to modify their functioning.^(61)^ Therefore, one might infer that authors intend to avoid bias in their studies. However, when one considers that the standard physical therapy practices in the ICU setting are similar to the ones reported in studies, the aforementioned assertions differ from Marcucci's view,^(62)^ according to whom physical therapy is complementary to palliative care, has a preventive nature, affords symptom relief and, whenever possible, provides patients an opportunity to develop and maintain their functional independence.

Safety criteria might go beyond the clinical and physiological ones, as shown in the present study. Restrictions in human and material resources might result in limitations to the mobilization of the critically ill, in addition to the particularities of each individual patient, which should always be emphasized. For EM to become essential and indispensable in the rehabilitation of the critically ill, professionals, physical therapists in particular, should be able to assess and suggest a safe treatment, adequate to the patient and duly monitored, so that the potential benefits of mobilization result in patient gains. For outcomes to be systematically favorable to patients, multidisciplinary staff members should have the required knowledge and be in continuous harmony.^(32)^

### Study limitations

To the best of our knowledge, the present is the first systematic review that analyzed the safety criteria used to start EM. However, as the study was based on the methods used in the analyzed studies, some limitations must be pointed out. First, as in any systematic review, there was potential for bias selection; however, we employed a broad-scoped search strategy so as to include the largest possible number of articles, analysis was independently performed by two reviewers and the exclusion criteria were clearly documented. Second, in some articles, the information was considerably limited (or provided substantially limited information on the methods used). Third, comparisons between studies were difficult due to the heterogeneity between samples and divergence in methods; the diversity of results, derived from the aims of each individual study, posed a true challenge to the present review. In addition, we should observe that the articles provided little information as to the occurrence of adverse events, which could have contributed to the interpretation of some data and helped readers in the choice of measures to adopt in clinical practice. These shortcomings stress the need for articles to include good descriptions of methods and information in general to facilitate reproducibility and the consolidation of the scientific evidence in this field.

## CONCLUSION

Cardiovascular criteria were the most frequently cited in the analyzed studies, exhibiting the largest number of variables. For respiratory criteria, the variables related to mechanical ventilation exhibited the highest concordance among authors. The authors considerably diverged in relation to neurological criteria, with lack of consensus mainly for assessment of the level of consciousness.

The present study reinforces findings reported in other studies on the criteria frequently used to ensure safety in the early mobilization of the critically ill, an approach currently growing in the intensive care setting in Brazil and abroad. The parameters and variables located in the present systematic review might be included in service routines so as to start, make progress and guide clinical practice.
